# Changes in Flight Altitude of Black-Tailed Gulls According to Temporal and Environmental Differences

**DOI:** 10.3390/ani14020202

**Published:** 2024-01-08

**Authors:** Jong-Hyun Park, In-Yong Jeong, Seung-Hae Lee, Jeong-Chil Yoo, Who-Seung Lee

**Affiliations:** 1 HAE-IN Ecological Research Institute, Busan 48304, Republic of Korea; skylovei-86@daum.net (J.-H.P.); haein@haeineco.kr (I.-Y.J.); jcyoo@khu.ac.kr (J.-C.Y.); 2 Department of Biology, Kyung Hee University, Seoul 02447, Republic of Korea; seunghae@kakao.com; 3 Environment Assessment Group, Korea Environment Institute, Sejong 30147, Republic of Korea

**Keywords:** black-tailed gull, flight height, home-range, GPS tracking

## Abstract

**Simple Summary:**

Gulls are known for their adaptability and can navigate various environments from coastal and inland areas to marine spaces. Despite extensive studies using GPS tracking devices to explore the habitat use and flight patterns of numerous gull and other bird species, little such research has focused on the widely-distributed black-tailed gulls (*Larus crassirostris*) inhabiting the Korean Peninsula. To fill this gap in our understanding, our study employed GPS trackers to analyze the flight behavior and habitat use of black-tailed gulls. Our results unveiled distinct ranges of activity and flight altitudes that were influenced by both season and region. These findings suggest that the flight behavior of black-tailed gulls is responsive to environmental changes. Our study contributes fundamental data on the flight behavior of black-tailed gulls.

**Abstract:**

In this study, GPS trackers were attached to black-tailed gulls (*Larus crassirostris*) breeding on five islands in Republic of Korea during April and May 2021, and their flight frequency, behavioral range, and flight altitude were compared during and after the breeding season. During the breeding season, the flight frequency was lowest on Dongman Island (28.7%), where mudflats were distributed nearby, and the range of activity was narrow. In contrast, it tended to be high on Gungsi Island (52%), where the breeding colony was far from land, resulting in a wider range of activity. Although the flight frequency on Dongman Island increased post-breeding season (42.7%), it decreased on other islands. The mean flight altitude during the breeding season was lowest on Dongman Island and highest on Napdaegi Island. In most breeding areas, the mean flight altitude during the post-breeding season was higher than that during the breeding season. However, the lead flight altitude was lower during the non-breeding season compared to that in the breeding season. The home range expanded after the breeding season, with no significant difference in lead time between the breeding and non-breeding seasons. Our findings reveal that black-tailed gulls exhibit varying home ranges and flight altitudes depending on season and geographical location. As generalists, gulls display flexible responses to environmental changes, indicating that flight behavior adapts to the evolving environment over time and across regions.

## 1. Introduction

Birds employ various flight strategies for each individual flight and consume a considerable amount of energy in the process [1]. Therefore, many bird species exhibit diverse flight patterns that are influenced by their primary activities, such as feeding, transporting food, moving between habitats, and migrating [1,2]. Gulls of the genus *Larus* are the top predators in marine ecosystems, showing adaptability by consuming various food sources and modulating their habitats according to food availability [3,4,5,6,7]. They, like other birds, employ flight strategies aimed at minimizing the energy required for flight, and these strategies are influenced by various factors, including environmental characteristics within their habitat, weather conditions, and the purpose of the flight (e.g., foraging, etc.) [1].

Studies on habitat use, migration, home ranges, and related movement patterns have been documented with advancements in GPS tracking technology [8,9,10]. GPS devices provide comprehensive information on flight behavior, including flight speed, altitude, and direction, resulting in numerous studies investigating the flight behavior of many species [11]. Gulls are among the birds that extensively utilize marine space, and there is active research employing GPS to analyze the impact of offshore wind power farm operations on their habitat usage and the potential for collisions [12,13,14].

Black-tailed gulls (*Larus crassirostris*) are a medium-sized species inhabiting East Asia. Approximately 1,000,000 black-tailed gulls exist [15], maintaining a stable population size [16]. However, this species is locally distributed in East Asia and may be vulnerable to habitat damage and rapid environmental change [17]. Black-tailed gulls breed on islands adjacent to the mainland of Republic of Korea, China, and Russia, as well as at locations tens of kilometers away [17,18,19,20,21]. In the Korean Peninsula, most breeding habitats are located along the western and southern coasts [18]. Studies have explored how these gulls use various environments such as coastal areas, mudflats, estuaries, and wetlands during the non-breeding season [21,22,23]. Black-tailed gulls are generalists that adapt their habitats and flight patterns in response to seasonal and regional changes [24,25].

Black-tailed gulls are the most common breeding birds along the west coast of the Korean Peninsula, and studies have explored their ecological characteristics. However, the majority of these studies were focused on aspects such as reproduction, kin recognition, and heavy metal contamination [21]. Recently, studies employing GPS trackers on black-tailed gulls have started to emerge, however, most of these studies primarily focus on migration routes and analysis of their home range [26,27,28,29,30]. However, as offshore wind farms are planned to be built in the waters around islands where this species breeds, information on their flight behavior and habitat use is needed to conserve their populations [31,32,33]. Therefore, in this study, we investigated the impact of seasonal and geographical changes on the flight altitude of black-tailed gulls from five breeding colonies in Republic of Korea by analyzing the relationship between flight altitude and speed using GPS trackers.

## 2. Materials and Methods

### 2.1. Study Sites

GPS tracking devices were deployed on 84 black-tailed gulls on five islands (i.e., five breeding colonies) situated along the west coast of Republic of Korea with the following distributions: 17 on Bulmugi Island, 16 on Dongman Island, 16 on Gungsi Island, 16 on Nando Island, and 19 on Napdaegi Island. Bulmugi Island (34°45′32″ N, 126°13′27″ E), located in the southwestern Yellow Sea, has an area of 32,590 m^2^, an altitude of 23 m, and is situated 4.7 km from the mainland. Herbaceous plants dominate the island, and its perimeter is encircled by rocks. Approximately 2700 pairs of black-tailed gulls breed on Bulmugi Island.

Dongman Island (37°32′42″ N, 126°16′21″ E), situated in the northwest of the Yellow Sea in Republic of Korea, covers an area of 82,314 m^2^. At an altitude of 93.9 m above sea level, it is the closest to the mainland among the surveyed areas at a distance of 3.0 km. The island is predominantly covered by woody plants at its center, while sand and mud flats are widespread. Most black-tailed gulls breed along the edges of islands [21].

Gungsi Island (36°40′02″ N, 126°02′06″ E), located in the middle-west of the Yellow Sea, has the largest area among the surveyed islands, covering 146,051 m^2^. It is 24 km from land at an altitude of 85 m above sea level. Herbaceous plants such as sedges and rapeseed dominate the island. Despite being uninhabited, Gungsi Island is frequently visited by people and among the surveyed locations is an area where black-tailed gulls have recently begun breeding.

Nando Island (36°39′36″ N, 125°49′25″ E), adjacent to Gungsi Island and located in the middle-west of the Yellow Sea, has an area of 47,603 m^2^ and is situated 27 km from land. The island’s altitude is 80 m above sea level, and while sandgrass dominates most of the island, cliffs characterize its edges. Designated and managed as a natural monument, Nando Island has limited access for people [21].

Napdaegi Island (35°15′57″ N, 126°13′17″ E), located in the southwest of the Yellow Sea, covers an area of 7645 m^2^ and is 8.0 km away from the mainland. Milsaceae dominate the island with scarce woody plants. The slope is gentle, resulting in less vegetation than that in other areas. Consequently, ample soil is exposed, providing breeding grounds for a small number of yellow-billed egrets and black-faced spoonbills.

### 2.2. Attaching GPS Trackers

To study the flight patterns of black-tailed gulls during a large portion of the breeding season, fieldwork was conducted at the breeding colony from 29 April to 5 May 2021 by capturing birds during the early incubation stage. The capture method involved the use of a claptrap, a discrete device consisting of a cloth-covered square steel frame equipped with a spring-trigger mechanism at its center. This trap is particularly suited for capturing nesting adult birds because of its small size and minimal noise production, ensuring minimal disturbance to nearby breeding black-tailed gulls.

Following capture, the black-tailed gulls were secured for the subsequent attachment of a solar-powered GPS tracker. The weight of the GPS tracker adhered to a critical guideline, recommended to be less than 3–5% of the bird’s body weight. To adhere to this recommendation, a 20 g GPS tracker was affixed to individuals weighing over 500 g, whereas a 10 g GPS tracker was attached to those weighing less than 500 g.

Following the method suggested by Thaxter et al. [34], which highlights a harness method using strings and hooks as a non-intrusive attachment technique that does not compromise survival and flight efficiency, GPS trackers were secured to the black-tailed gulls. Tubular Teflon strings and rings were employed in the harness method, with adjustments made for individual variations in shape and size.

The attached GPS tracker was configured to collect data at intervals of 30 min to 1 h, depending on the remaining battery level and time, and to transmit this information once a day. The collected data included location coordinates, altitude, speed, and time. To ensure the accuracy and reliability of the dataset, information recorded in regions where no black-tailed gulls typically travel, such as polar regions, flight altitudes exceeding 1000 m above sea level and speeds exceeding 100 km/h were excluded from the subsequent analysis. This methodological approach aimed to capture a representative dataset of flight behavior in black-tailed gulls during the breeding season while minimizing any potential adverse impacts on the study subjects.

### 2.3. Flight Behavior

The analysis used GPS coordinates collected from May to December 2021. The breeding season was defined as May, marking the initiation of breeding activities, until July, when black-tailed gulls typically vacate their breeding grounds. The post-breeding period was from August, following the completion of most breeding activities, to December, preceding their return to the breeding grounds.

To estimate space use, we used kernel density estimation (KDE) which is a method often used when analyzing tracking data, e.g., [8,10].

The speed at the time of data collection served as a key indicator for predicting bird behavior. Following the method proposed by Ross-Smith et al. [12], the analysis classified behaviors into three primary types: sitting, standing, or floating was recorded at <1 km/h; swimming or walking was recorded at 1–4 km/h; and flying was recorded at >4 km/h.

### 2.4. Statistical Analysis

The Shapiro–Wilk test was employed to assess the normal distribution assumption for all independent variables. Considering that each variable met the assumption of normal distribution, parametric tests were deemed appropriate for subsequent analyses. Factors influencing the flight altitude of black-tailed gulls were explored using generalized linear models (GLMs). Altitude served as the dependent variable when the birds were in flight and was defined as moving at a speed over 5 km/h. Flight speed, time (breeding and post-breeding seasons), and breeding colonies were designated as independent variables. Additionally, to investigate potential variations in flight speed between the breeding and post-breeding seasons due to brooding behavior, an interaction variable was included. The GLM analysis was conducted using the GLM package in R. Model selection was based on the corrected Akaike information criterion (AICc), and the model exhibiting the lowest AICc value was selected as the final model [35].

Home range was calculated using the kernel density estimation feature in the adehabitatHR R package [36].

A *t*-test was employed to compare differences in flight altitudes between the defined periods, and a one-way ANOVA test with post-hoc Tukey HSD was conducted to compare flight altitudes across different regions. R version 4.2.1 [37] was used for all statistical analyses. Results are presented as mean ± standard error.

## 3. Results

### 3.1. Flight Frequency

A total of 84 tracking devices were attached and 73 were used for analysis, excluding 16 tracking devices whose tracking period was too short due to death or interruption of reception, with the following distribution: 15 on Bulmugi Island, 14 on Dongman Island, 12 on Gungsi Island, 20 on Nando Island, and 13 on Napdaegi Island. Subsequently, 2,331,675 GPS data points were collected: 556,284 on Bulmugi Island, 242,169 on Dongman Island, 431,421 on Gungsi Island, 463,733 on Nando Island, and 638,068 on Napdaegi Island (Table 1).

**Figure 1:**
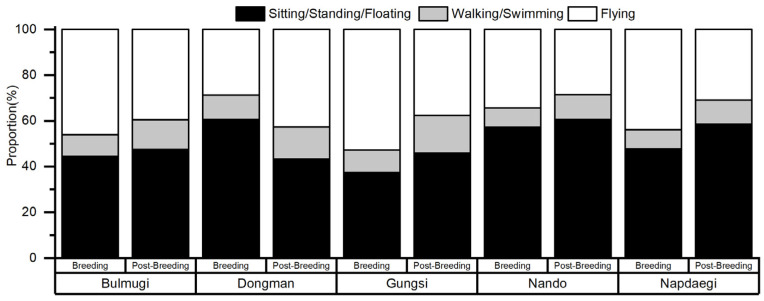
Differences in flight frequency of black-tailed gulls by period and region on five islands (Bulmugi, Dongman, Gungsi, Nando, and Napdaegi) on the west coast of Republic of Korea. Note that the breeding period was from May to July in 2021 and the post-breeding period was from August to December in 2021.

During the breeding season (May to July in 2021), flight frequency varied across the Islands. Gungsi Island had the highest frequency at 52%, followed by Bulmugi Island at 46.0%, Napdaegi Island at 43.8%, Nando Island at 34.2%, and Dongman Island at 28.7%. In the post-breeding season (August to December in 2021), Dongman Island had the highest flight frequency (42.7%), followed by Bulmugi Island (39.5%), Gungsi Island (37.6%), Napdaegi Island (30.96%), and Nando (28.6%) (Figure 1).

### 3.2. Model Selection

A GLM was used to analyze the effects of breeding colony, flight speed, and time on the flight altitude of black-tailed gulls. Eleven candidate models were selected for this study. Among these, the global model encompassing all independent variables and the model excluding flight speed, colony, and season exhibited the lowest AIC values.

When colony, speed, and season were excluded, there was no decrease in the model fit. However, in the model where variables representing interaction effects, such as colony × speed, colony × season, and season × speed were removed, the model’s explanatory power decreased significantly (Table 2).

The final model showed that breeding colony, season, and flight speed significantly influenced the flight altitude of black-tailed gulls. The flight altitude of the gulls was also positively correlated with flight speed. Interestingly, flight altitude during the post-breeding season was higher than during the breeding season (Table 3). There was a significant difference in flight altitude among breeding colonies. The flight altitude of gulls breeding on Bulmugi Island was higher than that on both Dongman and Nando Islands, but lower than the other islands (Gungsi and Napdaegi) (Table 3). There was a significant interaction between speed and season: the effect of speed during the breeding season was lower than the post-breeding season (Table 3). The flight altitude on Bulmugi Island was negatively correlated with that on Dongman Island (Table 3). The impact of season on flight altitude differed significantly across different breeding colonies: The flight altitude on Bulmugi Island was positively correlated with that on Dongman, Gungsi, and Nando islands, but negatively related to Napdaegi Island (Table 3). The flight altitude on four islands except Napdaegi Island during the post-breeding season was higher than during the breeding season, whereas the flight altitude on Napdaegi Island was higher during the breeding season (Table 3 and Table 4).

The mean flight altitude of black-tailed gulls breeding on Bulmugi Island was 33.80 ± 0.12 m; on Dongman Island, it was 25.70 ± 0.23 m; on Gungsi Island, it was 36.42 ± 0.15 m; on Nando Island, it was 29.63 ± 0.15 m; and on Napdaegi Island, it was 34.63 ± 0.11 m. The highest flight altitude was observed on Napdaegi Island, and Dongman Island showed the lowest flight altitude. During the post-breeding season, the mean flight altitude on each island was as follows: Bulmugi Island, 34.16 ± 0.14 m; Dongman Island, 34.20 ± 0.21 m; Gungsi Island, 38.10 ± 0.19 m; Nando Island, 39.97 ± 0.19 m; and Napdaegi Island, 31.56 ± 0.151 m (Table 4).

### 3.3. Flight Speed

**Figure 2:**
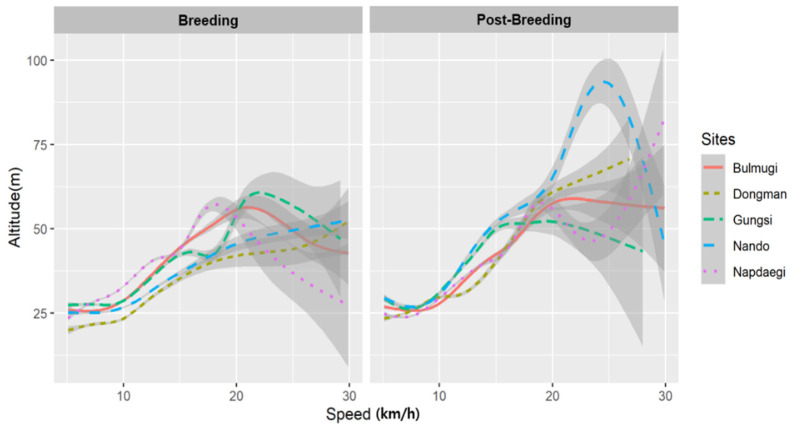
Relationship between flight altitude and speed of black-tailed gulls breeding on five islands (Bulmugi, Dongman, Gungsi, Nando, and Napdaegi) on the west coast of Republic of Korea. Note that the breeding period was from May to July in 2021 and the post-breeding period was from August to December in 2021.

During both the breeding and post-breeding seasons, flight altitude was positively related to flight speed (<15 m/h). However, during the breeding period, there was a tendency for altitude to decrease when flight speed exceeded 15 m/h. However, flight altitudes tended to decrease above 15 km/h during the breeding season, except on Nando Island. In the post-breeding season, the flight altitude of the gulls also tended to increase with flight speed, up to 15 km/h. Flight altitude showed a lower rate of increase at speeds up to 15 km/h, and the deviation was greater above 15 km/h, however, unlike during the breeding season, there was a tendency to fly at higher altitudes at speeds above 15 km/h. (Figure 2).

### 3.4. Comparison of Home Ranges between the Breeding and the Post-Breeding Seasons

**Figure 3:**
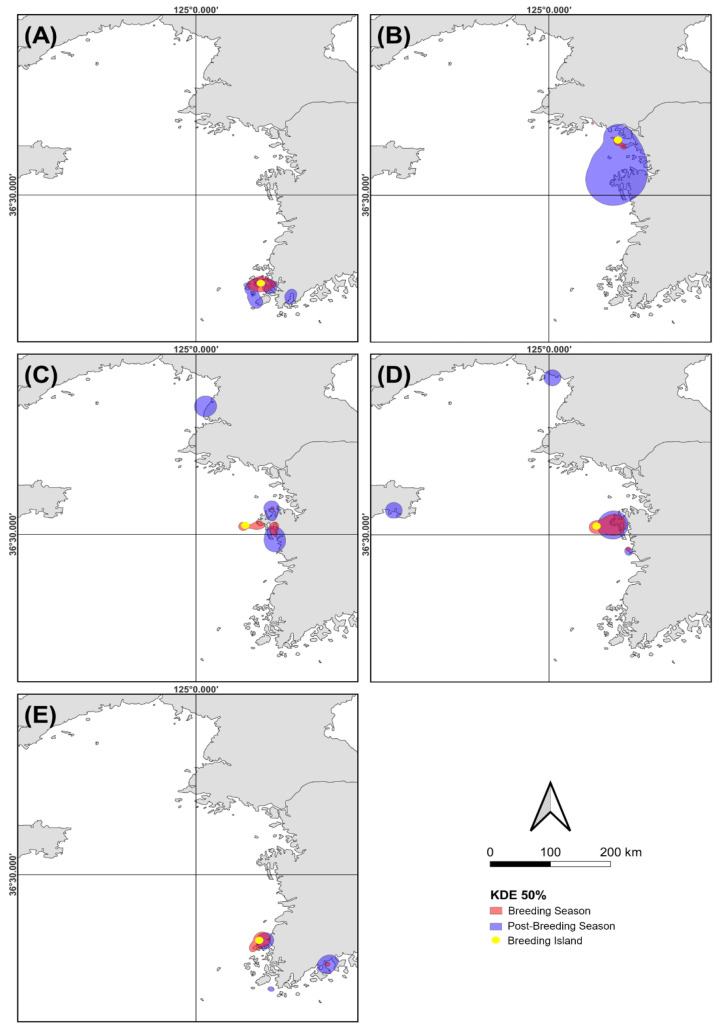
Kernel density estimates (KDE) at the 50% level for breeding and post-breeding seasons of black-tailed gulls on five breeding islands on the west coast of Yellow Sea in Republic of Korea: (**A**) Bulmugi, (**B**) Dongman, (**C**) Gungsi, (**D**) Nando, and (**E**) Napdaegi.

During the breeding season, most black-tailed gulls nested with their colonies around their breeding island. Post-breeding season, habitat preferences shifted to coastal areas and river estuaries adjacent to land (Figure 3). Black-tailed gulls breeding on Dongman Island exhibited a broader range of habitat usage, extending around the breeding colony as well as to the nearby sea after the breeding season (Figure 3).

As a core area, Kernel density estimation (KDE) at the 50% level indicates a tendency for the range of most black-tailed gulls breeding in specific grounds to expand. Every five Islands showed that the area during the non-breeding season was larger than during the breeding season (Figure 3): Bulmugi—1169.23 km^2^ during the breeding season and 2579.74 km^2^ during the post-breeding season, Dongman—320.41 km^2^ and 13,037.52 km^2^, Gungsi—957.26 km^2^ and 3666.66 km^2^, Nando—2080.71 km^2^ and 4084.76 km^2^, Napdaegi—1029.74 km^2^ and 1333.69 km^2^, respectively.

Overall, the core home-range (i.e., KDE 50%) on Dongman Island during the post-breeding season was significantly larger than during the breeding season, whereas there was no substantial difference in the range on Napdaegi Island between breeding and post-breeding seasons (Figure 3).

## 4. Discussion

On most of the breeding islands, the flight frequency of the black-tailed gulls tended to decrease during the post-breeding season (August to December, 2021); however, the flight frequency of the gulls breeding on Dongman Island increased during the period (Figure 1). During the breeding period (May to July, 2021), black-tailed gulls primarily rely on floating fish and squid as food sources around their breeding islands [38,39].

When black-tailed gulls utilize fish and squid as a foraging resource, the marine expanse is vast, and the distribution of food sources may be not uniform [40,41,42]. Therefore, the gulls use a broader area as a feeding ground compared to when they exploit coastal foraging sites, such as intertidal zones and harbors, and it might be better for the gulls to keep consistently flying over the sea for foraging. Moreover, the gulls it would be repetitive to walk or stand in order to forage shellfish, invertebrates and fishing byproducts in coastal areas (e.g., mudflats). Our results showed that the core home range of black-tailed gulls on most breeding islands was situated around the breeding colony and the coastal area near the breeding islands during breeding season (Figure 3).

However, at the end of breeding season (i.e., the post-breeding period), their activity range expanded, and they utilized the coast adjacent to the land as well as the sea. Therefore, the frequency of flights may decrease post-breeding season when they utilize not only the sea but also coastal areas, where they do not have to fly to search for food. In contrast, black-tailed gulls breeding on Dongman Island only used the surrounding mudflats and coastal area during the breeding season. However, their home range expands to the sea during the post-breeding season. Therefore, unlike other breeding islands where the proportion using the coast increases, the proportion of black-tailed gulls breeding on Dongman Island using marine space may increase at the end of breeding season. Our results suggest that this proportion would increase once the gulls complete their breeding and disperse/migrate to places where they spend time during the post-breeding period.

Gulls, including black-tailed gulls, are recognized for their generalist behavior and ability to adapt to various habitats based on food resource availability [43,44,45]. Black-tailed gulls exhibit flexibility in their foraging strategies, adapt to seasonal and environmental factors, and show diverse flight patterns corresponding to their habitats [24]. During their breeding period, many seabirds utilize foraging site close to their colonies [6,46]. In our study, 50% of the black-tailed gulls were located around the breeding islands (Figure 3). However, variations in mean flight altitude were observed among the breeding colonies. During the breeding period, the average flight altitude of black-tailed gulls breeding on Dongman Island was approximately 4 to 9 m lower than those breeding on other colonies (Table 3). This discrepancy could be attributed to differences in habitat. Our results also showed that the foraging site of black-tailed gulls on Dongman Island was concentrated around the breeding island and encompassed a relatively small area. In contrast, breeding populations at other colonies with higher mean altitudes extended their foraging site around the breeding grounds as well as both near and far along coasts adjacent to the land. Considering that birds generally use higher altitudes for long-distance movement [11], the flight altitude of black-tailed gulls on Dongman Island, where their core home range (i.e., 50% KDE) was narrow, might be lower than that at other breeding colonies. During the post-breeding period on Dongman Island, the mean flight altitude of black-tailed gulls was about 9 m higher than during the breeding season. However, the flight altitude of black-tailed gulls breeding on Napdaegi Island was about 3 m lower than that during the breeding season. The core home range of black-tailed gulls in most breeding areas, except Napdaegi, increased significantly; there was no significant difference in the range of population breeding on Napdaegi Island between the breeding and post-breeding periods. Consequently, it may be considered that the increase in the distance required to move between habitats due to the expanded area of action is the result of the increase in the mean flight altitude of black-tailed gulls.

The flight altitude of black-tailed gulls showed a positive correlation, with increasing flight speed up to ~15 km/h. However, beyond speeds of 15 km/h, there was no significant change, or even a decrease in flight altitude among most of the population (Figure 2). During the post-breeding season, there was a tendency for flight altitude to continue increasing, even at speeds exceeding 15 km/h. Our results support the theory that birds employ various flight strategies for efficient movement [1] by adjusting their speed and altitude based on geographical and environmental factors, such as weather, wind, and terrain, to minimize energy consumption during flight [2,47,48]. However, further study is necessary to understand the mechanism.

Black-tailed gulls are known for their relatively slow flight speeds [26]. Yoda et al. [26] characterized speeds higher than 15 km/h as indicative of flight to different areas rather than flight for feeding purposes. Based on the findings of this study, black-tailed gulls may migrate at altitudes ranging from 50 to 100 m, with no significant increase in flight altitude at speeds exceeding 15 km/h. Nevertheless, speeds above 15 km/h revealed variations in flight altitude during the breeding and post-breeding seasons, as well as in breeding areas, indicating substantial deviations. Consequently, the altitude of migratory flight is likely to vary depending on geographical and environmental factors.

Our results show differences depending on the season and breeding area in flight altitude however there was no dramatic change in flight altitude. Moreover, since weather conditions and individual differences were excluded from our analysis, further research is necessary to comprehend the flight behavior of black-tailed gulls.

## 5. Conclusions

Previous research on black-tailed gulls has predominantly concentrated on ecological aspects associated with breeding and behavior [49,50,51,52,53]. Consequently, there are comparatively few investigations into the habitat usage and flight behavior of this species. In our study, we verified that black-tailed gulls display distinct flight behaviors contingent on timing and environmental factors. Furthermore, the results of this study allow us to propose suggestions for minimizing the likelihood of bird collisions.

Among offshore wind power facilities, the area with the highest risk of bird collisions is the part of the blade that rapidly rotates due to the wind. Although there are variations among facilities, the blade typically rotates at an elevation of 30 to 250 m. In this study, the average flight altitude of black-tailed gulls in most breeding areas is 30 to 40 m. To reduce the possibility of collisions, it is essential to position the extreme end of the wind turbine rotor blade at an altitude higher than this. Additionally, considering that black-tailed gulls at all breeding sites extensively utilize the ocean around these areas during the breeding period, it is necessary to avoid installing offshore wind farms near breeding sites or to implement measures to prevent collisions.

## Figures and Tables

**Table 1:** Number of black-tailed gulls deployed with GPS trackers; breeding season tracking data for the five Korean islands from May to December 2021.

Breeding Colony	Number of Devices	Breeding	Post-Breeding	Total
Bulmugi	15	251,440	304,844	556,284
Dongman	14	116,721	125,448	242,169
Gungsi	12	245,455	185,966	431,421
Nando	19	217,430	246,303	463,733
Napdaegi	13	301,497	336,571	638,068
Total	73	1,132,543	1,199,132	2,331,675

**Table 2:** Comparisons of the better fitted models for factors affecting the black-tailed gull flight height on five islands (Bulmugi, Dongman, Gungsi, Nando, and Napdaegi) on the west coast (i.e., Yellow Sea) of Republic of Republic of Korea (2,331,675 GPS points from 73 individuals). Models are ranked based on differences in the Akaike’s Information Criteria (ΔAIC) and Akaike weights (AIC Wt). K is the number of estimated parameters.

Model	K	ΔAIC	AIC Wt
Colony + Season + Speed + Colony × Season + Season × Speed + Colony × Speed	7	0	0.143
Colony + Speed + Colony × Season + Colony × Speed + Season × Speed	6	0	0.143
Colony + Season + Colony × Season + Colony × Speed + Season × Speed	6	0	0.143
Season + Speed + Colony × Season + Colony × Speed + Season × Speed	6	0	0.143
Colony + Colony × Season + Colony × Speed + Season × Speed	5	0	0.143
Season + Colony × Season + Colony × Speed + Season × Speed	5	0	0.143
Colony + Colony × Season + Colony × Speed + Season × Speed	5	0	0.143
Colony × Season + Colony × Speed + Season × Speed	4	0	0.143
Colony + Season + Speed + Colony × Season + Season × Speed	6	146	0
Colony + Season + Speed + Season × Speed + Colony × Speed	6	2816	0
Colony + Season + Speed + Colony × Season + Colony × Speed	6	3011	0

**Table 3:** Summary of the generalized mixed models showing the highest correlation of the flight altitude of black-tailed gulls as a function of independent variables. Note that there were 2,325,427 GPS data points from 73 individuals.

Final Model	Estimate	Std. Error	t Value	*p*
Intercept	13.08	0.26	49.99	<0.01
Speed	1.75	0.24	71.48	<0.01
Season				
Breeding vs. Post-breeding	−2.80	0.24	−11.24	<0.01
Colony				
Bulmugi vs. Dongman	−3.23	0.44	−7.28	<0.01
Bulmugi vs. Gungsi	0.71	0.35	2.02	<0.05
Bulmugi vs. Nando	−3.09	0.36	−8.54	<0.01
Bulmugi vs. Napdaegi	0.75	0.32	2.32	<0.05
Speed × Season				
Breeding vs. Post-breeding	0.27	0.02	12.16	<0.01
Colony × Speed				
Bulmugi vs. Dongman	−0.24	0.04	−5.82	<0.01
Bulmugi vs. Gungsi	−0.05	0.03	−1.62	n.s
Bulmugi vs. Nando	0.04	0.03	1.40	n.s
Bulmugi vs. Napdaegi	0.23	0.03	7.47	<0.01
Colony × Season				
Bulmugi vs. Dongman	−5.27	0.23	22.88	<0.01
Bulmugi vs. Gungsi	−2.31	0.17	12.93	<0.01
Bulmugi vs. Nando	−5.90	0.19	31.04	<0.01
Bulmugi vs. Napdaegi	2.94	0.16	17.69	<0.01

**Table 4:** Mean flight altitude of black-tailed gulls breeding on five islands (Bulmugi, Dongman, Gungsi, Nando, and Napdaegi) on the west coast of Republic of Korea. Note that there were 2,325,427 GPS data points from 73 individuals. Data shown as mean ± SE (m). Using Tukey’s HSD test, superscript characters indicate a significant difference to the breeding islands identified by the character.

Season	Bulmugi (a)	Dongman (b)	Gungsi (c)	Nando (d)	Napdaegi (e)	*p*
Breeding	33.80 ± 0.12 ^b, c, d, e^	25.70 ± 0.23 ^a, c, e^	36.42 ± 0.15 ^a, b, d, e^	29.63 ± 0.15 ^a, c, e^	34.63 ± 0.11 ^a, b, c, d^	<0.01
Post-breeding	34.16 ± 0.14 ^c, e, d^	34.20 ± 0.21 ^c, e, d^	38.10 ± 0.19 ^a, b, d, e^	39.97 ± 0.19 ^a, b, c, e^	31.56 ± 0.15 ^a, b, c, d^	<0.01

## Data Availability

Data are contained within the article.
